# Data-driven fuzzy information granulation for predicting freight volume trends

**DOI:** 10.1371/journal.pone.0348239

**Published:** 2026-05-07

**Authors:** Yunbo Gao, Xinyu Wang, Ming Niu, Jianguo Li, Liping Cai, Rui Li

**Affiliations:** 1 College of Traffic and Vehicle Engineering, Wuxi University, Wuxi, China; 2 Freight Department, China Railway Lanzhou Group Co., Ltd., Lanzhou, China; 3 College of Materials Science and Engineering, Nanjing Forestry University, Nanjing, China; 4 College of Integrated Circuit Science and Engineering, Wuxi University, Wuxi, China; Universitas Mercatorum, ITALY

## Abstract

Rail freight volume trend prediction faces challenges due to data fuzziness, complexity and nonlinearity, and traditional deterministic prediction methods frequently fall short of practical application needs, particularly in addressing uncertainty. To overcome these limitations, we proposed a freight volume trend prediction model that integrated Fuzzy Information Granulation (FIG) with evolutionary optimization. The three-phase methodology establishes: (1)A FIG method was utilized to transform raw time-series into tri-granular representations (Low, R, Up) through fuzzy c-means clustering with temporal constraints, extracting feature information from the raw time-series data and encapsulating it into information granules (2) For complex predictions with small samples, we applied a Support Vector Machine (SVM) for granular modeling, combined with an Improved Particle Swarm Optimization (IPSO) algorithm featuring dynamic inertia weights and mutation operators to prevent premature convergence during training. (3) A hybrid FIG-IPSO-SVM architecture implementing granular-level regression with uncertainty quantification. Validation using 9-year operational records (2013–2022) from the Lanzhou Freight Center (n = 114 monthly observations) in China reveals statistically significant enhancements: compared to the FIG-GS (grid search)-SVM and FIG-PSO (Particle Swarm Optimization)-SVM algorithms, the proposed IPSO-SVM algorithm achieved the smallest prediction error for each granulated set (Low, R, Up) and the smallest mean maxima of absolute percentage error (${APE}_{\text{M}}$) for the prediction interval of freight volume, at 5.03%. Moreover,it yielded the tightest prediction interval, characterized by a relative width (*R*_*w*_) of just 8.53% and a corresponding interval width (*W*) of only 516,209 tons, surpassing all benchmark models.These findings validate that the FIG-IPSO-SVM framework substantially improves interval prediction precision and trend detection reliability, providing actionable intelligence for railway infrastructure planning and operational optimization.

## 1. Introduction

Rail freight transportation serves as a vital component for national economic development [1]. However, its share of the total societal freight volume has been on a downward trend in recent years. Amidst the current impetus for the “Belt and Road” initiative, rail freight encounters both substantial opportunities and significant challenges [2,3]. The initiative has spurred cross-border freight demand and optimized the layout of railway networks, yet it also imposes higher requirements on the accuracy and timeliness of freight volume forecasting. Forecasting rail freight volume has become a critical research focus, as accurate predictions of its intervals and trends are instrumental in adjusting transportation structures, optimizing resource allocation, and planning regional economic growth [4]. It can directly guide the allocation of rolling stock, the scheduling of transportation routes, and the investment in infrastructure construction, thereby reducing operational costs and improving transportation efficiency. However, due to the ambiguity, complexity, and nonlinearity inherent in rail freight volume data, driven by factors such as macroeconomic fluctuations, industrial structure adjustments, and policy changes, traditional prediction methods frequently fail to meet the requirements of accuracy in practical applications [5,6]. Consequently, identifying an efficient and precise prediction method has become an urgent priority in the field of freight volume forecasting. Currently, the prediction methods for railway freight volume are mainly categorized into two groups: qualitative and quantitative analysis.

Qualitative analysis employs expert judgment, based on a thorough examination of the railway freight system, to assess future freight volume trends [7]. Common qualitative analysis methods include the expert opinion method, Delphi method [8], subjective probability method [9], and analogical inference method [10]; however, their results are heavily influenced by subjective factors, leading to low reliability in scenarios with complex data characteristics. Consequently, many studies use qualitative methods as auxiliary tools to complement quantitative methods.

Quantitative analysis methods [11], which rely on historical data and mathematical models, predict future freight volumes through calculation and statistical analysis, offering more precise and verifiable forecasting outcomes.Common quantitative analysis methods for freight volume include linear regression analysis, neural networks, and gray prediction models. To analyze the impact of various factors on railway freight volume, linear regression constructs a model of the linear relationship between independent and dependent variables [12]. The primary regression method used for predicting freight volume is currently multiple linear regression [13,14]. However, for complex nonlinear variable predictions, the accuracy of linear regression analysis may not meet practical needs, as it cannot capture the nonlinear correlations between freight volume and its influencing factors. In contrast, neural network is a universal approximator that can better learn and approximate any nonlinear relationships [15], thus performing better when dealing with highly nonlinear relationships between variables. Methods used for freight volume prediction include Back Propagation Neural Network (BPNN) [16], Radial Basis Function (RBF) neural network [17], Long Short-Term Memory(LSTM) [18], Convolutional Neural Network (CNN) [19,20], and deep learning (DL) [21]. However, due to risk minimization criteria [22], neural network methods demand more data and computational resources during the training process, and insufficient data compromise prediction accuracy. their training process requires more data and computational resources, and insufficient data may affect prediction accuracy. In contrast, gray models are well-suited for predictions with limited data, owing to their method of accumulating original data to generate new series and establishing differential equations. Such models have been applied in railway freight volume prediction [23]. To leverage the complementary strengths of neural networks and gray models, some studies have proposed hybrid frameworks integrating the two approaches [24]. However, it is worth noting that while gray models can handle small sample data, they are not suitable for situations requiring complex models, as they struggle to capture high-dimensional nonlinear relationships in data.

The Support Vector Machine (SVM) algorithm is not only adept at forecasting small sample data sets [25], but also outperforms the aforementioned gray model methods in complex predictions involving small samples, where the latter often fail to capture high-dimensional nonlinear relationships. Moreover, SVM methods boast a simple structure, robust noise resistance, and excellent generalization capabilities, making them widely used in time series prediction [26,27]. Consequently, SVM is highly effective for predicting complex railway freight volumes. [28]. However, the predictive performance of SVM is heavily dependent on the optimization of parameter selection [29], and traditional parameter selection methods such as grid search (GS) demand excessive computational resources, and are prone to falling into local optima. Although Particle Swarm Optimization (PSO) mitigates this issue [30], its tendency to converge prematurely limits global optimization [31,32]. An Improved Particle Swarm Optimization (IPSO) was introduced to improve SVM’s global prediction accuracy. The proposed algorithm achieves balanced exploration-exploitation through adaptive adjustment of inertia weights integrated with dynamically optimized learning factors, effectively addressing the premature convergence problem of traditional PSO.

However, the aforementioned methods yield deterministic freight volume forecasts, but fail to capture the trends and fluctuations in freight volume changes. The system for predicting railway freight volume is inherently dynamic and time-dependent, exhibiting a degree of uncertainty and random variations. For railway department managers, the ability to anticipate the future range and trend of freight volume changes would significantly aid in planning and management, ultimately leading to cost savings and enhanced efficiency. Deterministic predictions can only provide a single value, which is insufficient to support risk assessment and decision-making under uncertain conditions. To address this need, Fuzzy Information Granulation (FIG), a data preprocessing technique designed to manage uncertainties, can be employed. By categorizing data into granulated sets [33], FIG simplifies data analysis and improves model interpretability, while preserving the volatility characteristics of time series data.Following FIG preprocessing, predictions are made based on the granulated sets, enabling the forecasting of both the interval and trend of freight volume changes. This approach not only streamlines sample data but also preserves crucial characteristic information within the dataset, and FIG demonstrated its effectiveness in a series of experiments in the area of transport [34].

This study employed the FIG-IPSO-SVM method to forecast freight volume trends. The proposed FIG-IPSO-SVM framework is designed to address three fundamental challenges in railway freight volume forecasting: uncertainty, nonlinearity, and small-sample complexity. The methodology was applied to monthly freight-volume time series from the Lanzhou Freight Center in China. Specifically:

(1) FIG is employed to manage data uncertainty and volatility. By transforming raw time-series into tri-granular sets (Low, R, Up), FIG retains essential trend information while reducing noise and complexity, thereby providing a robust basis for interval prediction.
(2) IPSO is then employed to adaptively optimize SVM hyperparameters by adjusting inertia weights and learning factors, which overcomes the premature convergence limitations of standard PSO and thus enhances the prediction accuracy and robustness of the model.
(3) The optimized SVM finally executed granular-level regression for freight volume trends prediction. This choice is based on SVM is selected for its strong generalization capability in small-sample settings and ability to model nonlinear relationships via kernel functions, making it suitable for complex, small-scale freight data.

This integrated framework proves superior in handling short-term complex freight volume trends by enabling granular-level interval forecasting—which quantifies both trend and uncertainty beyond deterministic models—as validated by case studies, and it provides robust support for enhancing railway management efficiency and resource allocation.

## 2. Trend prediction model based on FIG-IPSO-SVM

### 2.1. Fuzzy information granulation

Information Granulation, initially introduced by Zadeh L.A. [35], is based on the principle of partitioning a whole into segments for analytical purposes, guided by specific criteria. These segments are aggregated either due to challenges in differentiation, inherent similarities, or functional coherence, each constituting an information granule. FIG encapsulates these granules within a fuzzy set framework.

For time-series $X=(x_{1},x_{2},\ldots ,x_{n})$,the process of fuzzy granulation can be accomplished by constructing fuzzy particles *P* on *X*. This necessitates the establishment of a fuzzy concept *G*– a fuzzy subset of the universe X− that aptly encapsulates the inherent characteristics of *X*. Upon defining the fuzzy concept *G* through its membership function *f*, the associated fuzzy particle *P* is concurrently delineated. The formula for determining FIG is as Eq. (1):


g≜x is Gx∈X (1)

Common basic forms of fuzzy particles include triangular, trapezoidal, Gaussian, and parabolic. This paper adopted the triangular fuzzy particle, whose membership function is:


$$f(x,a,m,b)=\left\{ \begin{array}{cc} 0, & x<a\\ \frac{x-a}{m-a}, & a\leq x\leq m\\ \frac{b-x}{b-m}, & m<x\leq b\\ 0, & x>b \end{array}\right.$$ (2)

where *a*, *m*, and *b* represent the three characteristic parameters of the FIG. They correspond to the minimum value (also referred to as the lower bound), the average value (also known as the median), and the maximum value (also termed as the upper bound) within each granulation window for the changes in the original data.

The FIG methods primarily encompass two key processes: partitioning windows and fuzzification. Partitioning windows entails segmenting the time series data into several smaller, manageable blocks, which function as operational windows. Fuzzification, on the other hand, involves converting each of these windowed data segments into a fuzzy set. This study employed the granulation method developed by W. Pedrycz.

### 2.2. Support vector machine

SVM is a sophisticated machine learning technique rooted in the principles of statistical learning, renowned for its robust intelligent learning capabilities [26]. Grounded in the concepts of structural risk minimization and the Vapnik-Chervonenkis (VC) dimension theory, SVM excels at striking an optimal balance between model complexity and its ability to generalize training data, thereby exhibiting remarkable predictive accuracy and broad applicability. It demonstrates substantial benefits in scenarios involving limited datasets, non-linear relationships, as well as intricate classification and regression challenges [36].

The fundamental methodology for SVM to tackle non-linear regression issues is rooted in the Mercer kernel expansion theorem. By employing a non-linear mapping function, denoted as f(·), the input space undergoes transformation into a higher-dimensional linear feature space, also known as the Hilbert space. Subsequently, within this elevated feature space, a linear model is constructed with the purpose of approximating the underlying regression function.


f(x)=ω·ϕ(x)+b (3)

In Eq. (3), ω represents the generalized parameters of the function, while *b* denotes the offset. This paper employs ε-SVM, where ε signifies a predetermined insensitivity loss value; that is, if the discrepancy between the predicted and actual values does not surpass ε, it is deemed that there is no prediction loss. The optimization problem can thus be articulated as follows:


$$\min_{\omega ,b,\xi }\frac{1}{2}\|\omega {\|}^{2}+C\sum_{i=1}^{n}(\xi_{i}+\xi_{i}^{*})$$ (4)

$$\text{s.t.}\left\{ \begin{array}{c} y_{i}-\omega \cdot \phi (x_{i})-b\leq \varepsilon +\xi_{i}\\ \omega \cdot \phi (x_{i})+b-y_{i}\leq \varepsilon +\xi_{i}^{*}\\ \xi_{i},\xi_{i}^{*}\geq 0,\;i=1,2,\cdots ,n \end{array}\right.$$ (5)

In the formula, *C* > 0 is the penalty factor, which controls the degree of punishment for samples that exceed the error ε, thereby balancing the complexity of the model and the training error. $\xi_{i}$,$\xi_{i}^{*}$ are the slack variables, reflecting the extent to which samples deviate from the regression model, thus enhancing the model’s generalization ability and adaptability to noisy data.

By employing the Lagrange method, the original optimization problem is converted into its corresponding dual problem.


$$\min\frac{1}{2}\sum_{i,j=1}^{n}(\alpha_{i}-\alpha_{i}^{*})(\alpha_{j}-\alpha_{j}^{*})K(x_{i},x_{j})+\varepsilon \sum_{i=1}^{n}(\alpha_{i}+\alpha_{i}^{*})-\sum_{i=1}^{n}y_{i}(\alpha_{i}-\alpha_{i}^{*})$$ (6)

$$\text{s.t.}\left\{ \begin{array}{c} \sum_{i=1}^{n}(\alpha_{i}-\alpha_{i}^{*})=0\\ \alpha_{i},\alpha_{i}^{*}\in [0,C] \end{array}\right.$$ (7)

In the formula, $\alpha_{i}$ and $\alpha_{i}^{*}$ represent Lagrange multipliers, while $K(x_{i},x_{j})=\phi (x_{i})\phi (x_{j})$ defines the kernel function. This kernel function facilitates a nonlinear transformation that maps the input space into a linear higher-dimensional space [37]. Given the superior nonlinear prediction performance of the RBF and its requirement to adjust fewer parameters, this study selected the RBF as the kernel function. The RBF can be mathematically represented as Eq. (8):


$$K(x_{i},x_{j})=\exp\left(\frac{-\|x_{i}-x_{j}{\|}^{2}}{2\sigma^{2}}\right)$$ (8)

In the equation, σ signifies the bandwidth of the kernel function, and *x*_*i*_ corresponding to $\alpha_{i}-\alpha_{i}^{*}=0$ is the Support Vector (SV). The regression function obtained is shown in Eq. (9)


$$f(x)=\sum_{x_{i}\in SV}(\alpha_{i}-\alpha_{i}^{*})K(x_{i},x_{j})+b$$ (9)

From the aforementioned formula, it is evident that the computational complexity of the model is no longer constrained by the number of samples but instead depends solely on the quantity of SV within those samples, thereby significantly reducing the computational burden of the model. Samples that are not SVs do not affect the model construction process—a characteristic that indirectly enhances SVM’s adaptability to noisy data. Since the predictive performance of SVM relies on the selection and tuning of its parameters and kernel functions, optimizing the hyperparameters (C,σ) is crucial [38].

**Fig 1:**
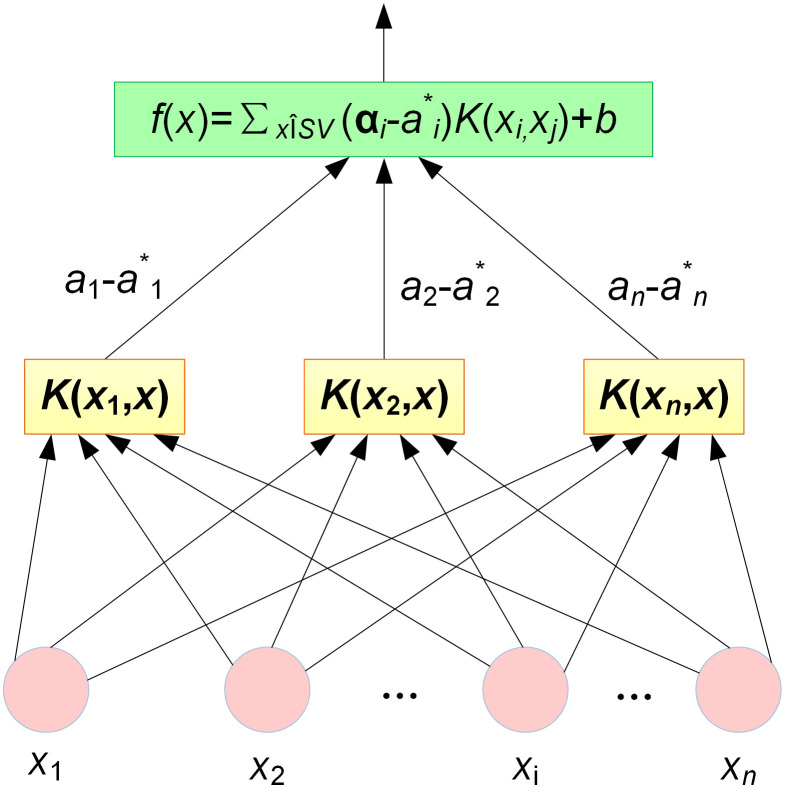
The architecture of the SVM regression model.

The architecture of the SVM regression model is illustrated in Fig 1, embodying a three-tiered neural network layout [39], where the input and output layers of a SVM learning machine are connected via kernel function nodes. Each kernel function node is a support vector, and the SVM output is generated through a linear combination of these kernel function nodes. The efficacy of SVM predictions hinges predominantly on two key parameters: the regularization parameter “*C*” and the “σ” parameter of the RBF kernel. To enhance both precision and generalizability in the SVM regression framework, this study employs the IPSO algorithm to optimize these critical hyperparameters (*C*, σ).

### 2.3. Improved particle swarm optimization algorithm

PSO algorithm was proposed by Eberhart and Kennedy in 1995 [35]. It is a population-based optimization algorithm based on swarm intelligence. The basic idea of the PSO algorithm is to simulate the foraging behavior of birds [40], where each potential solution is treated as a “particle” in the search space. The particles update their positions and velocities based on their own experience and those of other particles in the group, aiming to gradually approach the global optimum [41].

In the *k*-th iteration within a *d*-dimensional space, particle *i* computes and updates its velocity (*V*) and position (*X*) by tracking two critical values: the optimal position discovered by the particle itself $(P_{id}^{k}$), and the most favorable position identified by the entire swarm to date $(P_{gd}^{k}$). In other words,


$$V_{id}^{k+1}=\omega \times V_{id}^{k}+c_{1}\times r_{1}\times (P_{id}^{k}-X_{id}^{k})+c_{2}\times r_{2}\times (P_{gd}^{k}-X_{id}^{k})$$ (10)

$$X_{id}^{k+1}=X_{id}^{k}+V_{id}^{k+1}$$ (11)

where ω represents the inertia weight, which dictates the impact of historical velocity; *c*_1_ and *c*_2_ are the individual and social learning factors, parameters employed to modulate the relative significance of *P*_*id*_ and *P*_*gd*_; *r*_1_ and *r*_2_ are two uniformly distributed random numbers within the interval [0,1], utilized to enhance the randomness of the search.

Despite its advantages, the PSO algorithm encounters certain challenges, including tendencies towards premature convergence and suboptimal solution quality. To mitigate these issues, this paper introduces an enhancement strategy involving the adaptive tuning of PSO parameters (ω, *c*_1_, and *c*_2_).


$$\left\{ \begin{array}{c} \omega =\omega_{\text{max}}-(\omega_{\text{max}}-\omega_{\text{min}})(\frac{k}{T_{\text{max}}}{)}^{2}\\ c_{1}=c_{1\text{max}}-(c_{1\text{max}}-c_{1\text{min}})(\frac{k}{T_{\text{max}}})\\ c_{2}=c_{2\text{min}}+(c_{2\text{max}}-c_{2\text{min}})(\frac{k}{T_{\text{max}}}) \end{array}\right.$$ (12)

where *T*_*max*_ signifies the maximum number of iterations; the ranges for the inertia weight and learning factors are as follows: $\omega \in [\omega_{min},\omega_{max}]$, $c_{1}\in [c_{1min},c_{1max}],$ and $c_{2}\in [c_{2min},c_{2max}].$

### 2.4. Methodological rationale and framework robustness

The selection of the analytical approaches—FIG, SVM, and IPSO—was driven by the need to address three distinct characteristics of railway freight volume data: inherent fuzziness, evident nonlinearity, and limited sample size. To systematically tackle these challenges, each component of the framework was chosen for its specific strengths:

First, Fuzzy Information Granulation (FIG) was employed to handle the fuzziness and noise in the time-series data. It transforms raw, ambiguous data into structured granules (Low, R, Up), thereby distilling underlying trend features while concurrently quantifying uncertainty.

Second, Support Vector Machine (SVM) was selected to manage the nonlinearity and limited sample size. It offers established efficacy in small-sample regression and can model complex nonlinear relationships through kernel function mapping, without requiring extensive data.

Third, the Improved Particle Swarm Optimization (IPSO) algorithm was adopted to optimize SVM hyperparameters. It outperforms standard PSO and grid search by utilizing an adaptive inertia weight and mutation mechanism, which effectively mitigate premature convergence and enhance global search efficiency in high-dimensional spaces.

Recognizing that railway freight volumes are influenced by various external and often unobserved factors—such as seasonal demand cycles, macroeconomic shifts, and industrial policy changes—the proposed framework is designed to inherently account for these confounding influences, despite their explicit modeling falling outside the scope of this trend-focused study. This robustness is achieved through three integral mechanisms:

First, temporal and seasonal confounders are directly addressed within the FIG process. By employing fixed-width temporal windows (e.g., 3 months), local seasonal and cyclical patterns are encoded into the tri-granular representations (Low, R, Up).

Second, the model’s capability to capture complex, nonlinear interactions is fortified by the SVM’s kernel-driven mapping. This allows the model to approximate intricate relationships between historical data and future trends without requiring explicit input of confounding variables.

Third, the uncertainty attributable to unobserved or unmodeled factors is formally quantified through the prediction intervals generated by granule-level regression. This provides a transparent and robust measure of forecast uncertainty.

In summary, this methodology focuses on extracting predictive patterns directly from the historical time series, while systematically acknowledging and quantifying the uncertainty introduced by potential confounding factors.

### 2.5. FIG-IPSO-SVM based freight volume trend prediction

**Fig 2:**
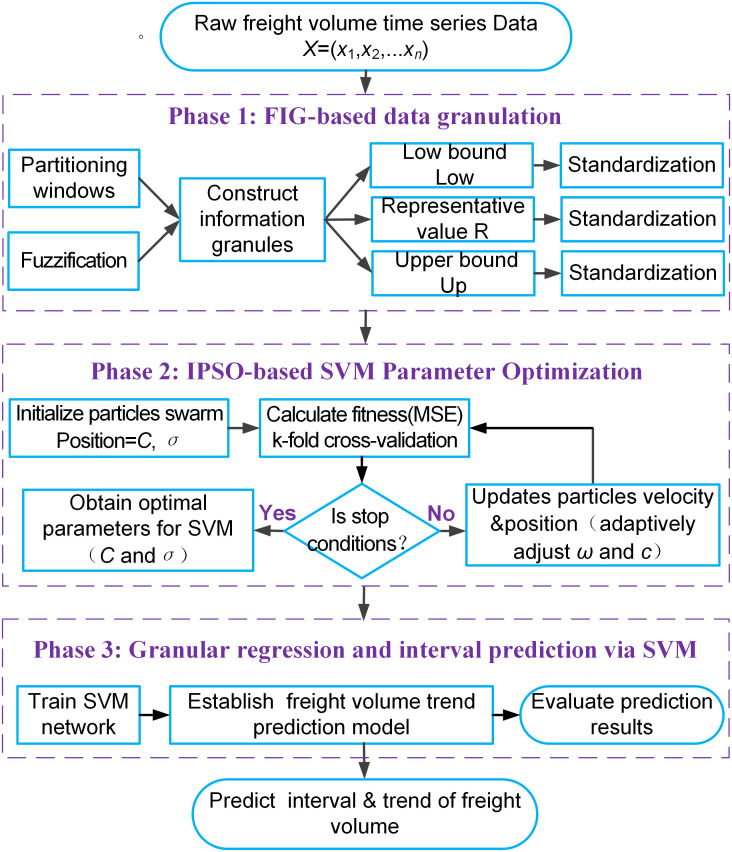
Flowchart of the freight volume trend prediction model based on FIG-IPSO-SVM.

The proposed freight volume trend prediction model integrates FIG, IPSO, and SVM into a coherent three-phase framework, as illustrated in Fig 2. This hybrid architecture is specifically designed to transform raw, uncertain time-series data into actionable interval forecasts that capture both central trends and inherent volatility.

#### 2.5.1. Phase 1 FIG-based data granulation.

The raw monthly freight volume time series $X=(x_{1},x_{2},...,x_{n})$ is processed using FIG to manage its inherent fuzziness and complexity. It is partitioned into windows, and each window is fuzzified into three key information granules from the raw data: the lower bound (Low), the representative central value (R), and the upper bound (Up). Subsequently, the data within each granulation set was standardized to a common scale for subsequent regression prediction using IPSO-SVM.

#### 2.5.2. Phase 2 IPSO-based SVM parameter optimization.

In this IPSO–based SVM parameter optimization process, each granule set is standardized before being fed into a SVM model, whose predictive performance largely depends on its hyperparameters *C* and σ of RBF. These parameters are optimized using IPSO. Initially, the particle swarm is randomly initialized with each particle’s position representing a candidate (C,σ) pair, and the fitness of each particle is evaluated using the mean squared error (MSE) derived from k–fold cross–validation to mitigate overfitting and enhance generalization. The swarm then iteratively updates the velocity and position of each particle according to Eq. 10 and 11, while adaptively adjusting the inertia weight ω and learning factors *c*_1_ and *c*_2_ based on Eq. 12 to dynamically balance global exploration and local exploitation. The optimization continues until a stopping criterion is met–either when the maximum number of iterations is reached or a preset precision threshold is achieved–at which point the optimal parameter set (C,σ) is obtained for training the final SVM model; otherwise, the iterative search proceeds.

#### 2.5.3. Phase 3: Granular regression and interval prediction via SVM.

Using the optimal parameters (C,σ) obtained in Phase 2, three separate SVM networks are trained on the granulated Low, R, and Up sets, thereby establishing a freight volume trend prediction model. The model’s performance is evaluated by comparing the predicted freight volumes with the actual observed values. For future time steps, the three trained SVMs simultaneously generate corresponding predictions, which together form a predictive interval and depict the likely trend trajectory. This interval quantitatively captures the expected range of future freight volumes, offering decision-makers not only a most-probable trend projection but also an explicit measure of forecast uncertainty.

The SVM regression model was implemented in MATLAB (R2021b, MathWorks) using the LIBSVM toolbox [42]. The parameter optimization procedure (psoSVMcgForRegress.m) was adapted from the open-source code accompanying Wang et al. [43], with modifications to the inertia weight ω and the learning factors *c*_1_ and *c*_2_ as described in Eq.12 in Section 2.3. The core structure of the algorithm, including the velocity and position update rules, remains unchanged.

## 3. Case simulation and analysis

### 3.1. Processing of raw freight volume data using FIG

The dataset employed in this study was sourced from the Lanzhou Freight Center, China Railway Lanzhou Group Co., Ltd. This data originates from the official operational statistics of the freight center, which is a key railway logistics hub in northwestern China. The dataset spans 114 consecutive months (July 2013–September 2022), which was selected for its representative freight volume trends and data completeness. It was a sufficiently long time series for capturing seasonal, cyclical, and trend components while avoiding outdated economic patterns.

**Fig 3:**
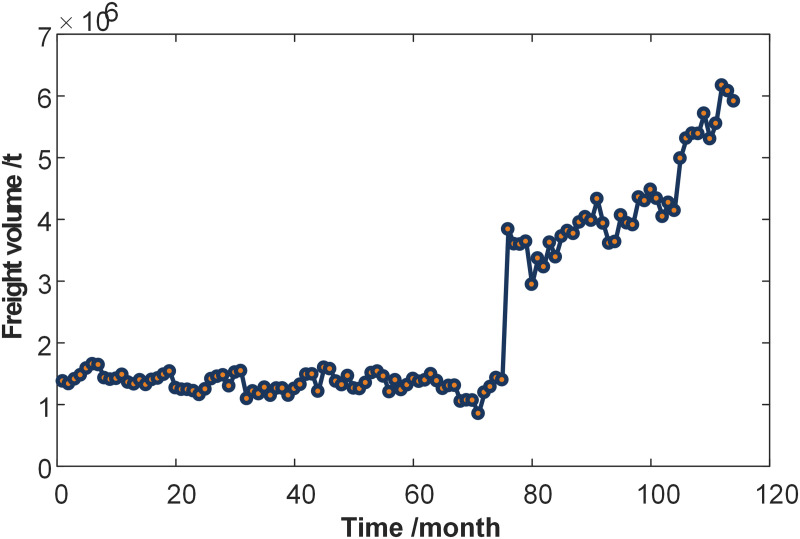
Raw freight volume time series data.

Prior to granulation, the raw monthly freight volume series underwent systematic quality checks. No missing values were found within the 114-month study period. The raw freight volume series data is illustrated in Fig 3 Furthermore, an Augmented Dickey-Fuller (ADF) test was subsequently conducted, yielding a *p*-value of 0.22 (ADF statistic = −2.15), which exceeds the 0.05 significance threshold. This result confirms the series’ non-stationarity and inherent trend, thereby justifying the application of trend-sensitive granulation and modeling approaches.

The final three months (October–December 2022) were reserved as a hold-out validation set to assess model generalization, consistent with common practice in time-series forecasting where recent data is used for testing. Accordingly, the training set comprised the first 111 months of data, to which FIG was applied for feature extraction and uncertainty modeling.

**Fig 4:**
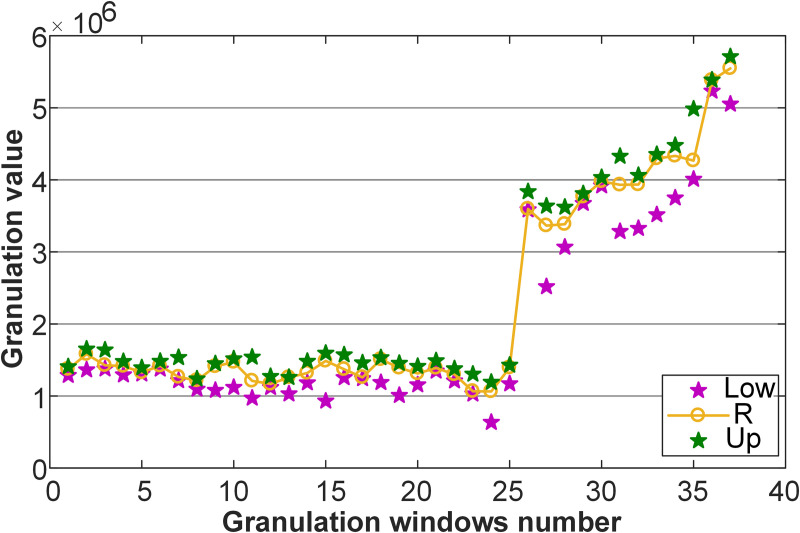
Granules of freight volume.

Fig 4 illustrates the granules of freight volume, from which both the morphology and distribution were clearly discernible. This step offered a more refined and effective data foundation for subsequent interval prediction.

### 3.2. IPSO-SVM-Based Freight Volume Trend Modeling

Afterwards, IPSO-SVM was utilized for regression and prediction of the granules. The three outputs, namely, Low, R, and Up, were treated as training dataset for model development.

Firstly, the IPSO optimization algorithm was employed to select the important parameters *C* and σ for the SVM model. The initial parameter settings for IPSO are shown in Table 1. After iterative optimization, the SVM model parameters for each granulated set obtained, and the results of SVM based on granules are presented in Table 2.

**Table 1:** Initial parameter settings for IPSO.

Parameters	Particle number	Maximum iterations	*C*	σ	ω	$c_{1}$	$c_{2}$	k
Values	20	200	[0.1, 100]	[0.01, 1000]	[0.8, 1.2]	[0.5, 2.5]	[0.5, 2.5]	5

**Table 2:** Results of SVM based on granules.

Granulation set	Optimized parameters (*C*, σ)	Training results (*r*^2^)
Low	(35.6133, 0.0642)	0.9603
R	(22.4301, 0.1573)	0.9367
Up	(69.3998, 0.1863)	0.9585

**Fig 5:**
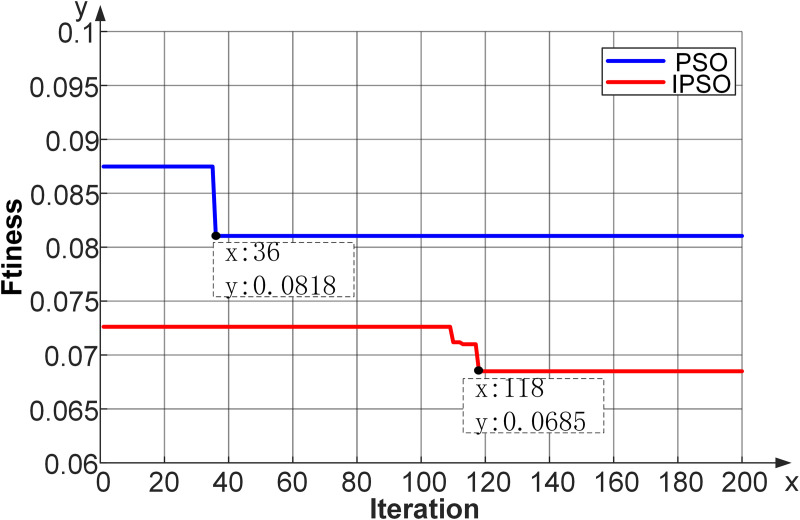
Comparison of fitness between the convergence curves based on PSO and IPSO.

A comparative analysis of fitness convergence curves between IPSO and PSO is illustrated in Fig 5. The IPSO algorithm achieved a 16.26% reduction in fitness value compared to PSO (0.0685 vs. 0.0818), with required iterations of 118 and 36, respectively. The results demonstrate that the proposed IPSO effectively mitigates the premature convergence issue inherent in PSO, indicating that IPSO is more suitable for parameter optimization in SVM models.

**Fig 6:**
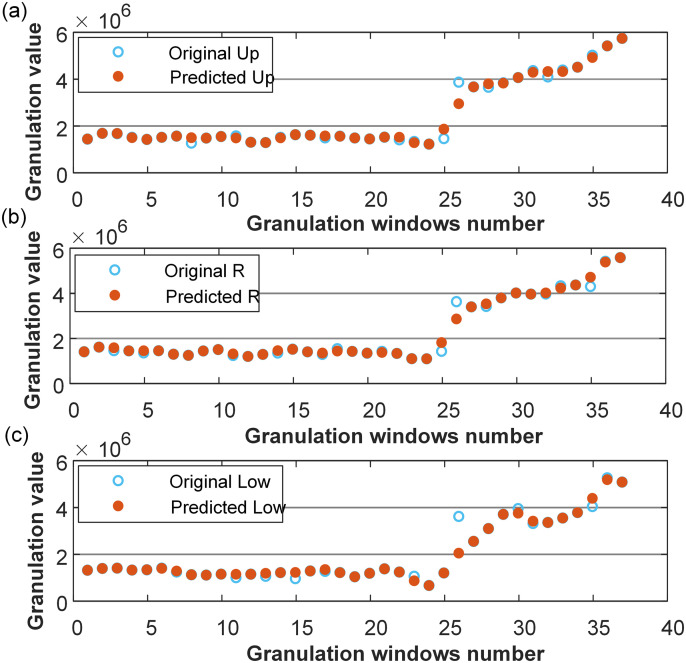
Prediction results for granulated sets: (a) Up, (b) R, (c) Low.

Then, the optimized parameters were applied to establish the SVM model, with the prediction square correlation coefficient *r*^2^ for model training being close to the maximum value of 1, and the minimum value also reaching as high as 0.9367. The prediction results for the granulated sets (Low, R, and Up) are shown in Fig 6, clearly demonstrating a high level of consistency between the predicted values and the actual values for each granulated set. As shown in Table 2 and Fig 6, the IPSO-SVM model demonstrated excellent modeling performance and high accuracy.

**Fig 7:**
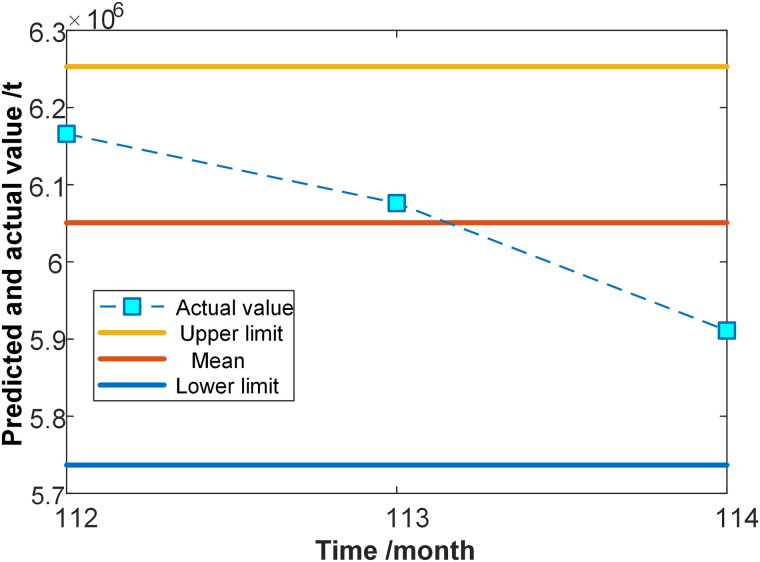
The forecasted interval of freight volume for the left data.

The forecasted interval of freight volume for the left data spanning October, November, and December is illustrated in Fig 7. The actual freight volume figures from October to December revealed that the actual values of freight volume in the following period reside between the upper and lower limits of the trend, with a distribution around the average. This indicated that the predicted interval of freight volume changes for the upcoming period was accurate.

In addition, the last set of data processed by FIG is [Low, R, Up]= [5051977, 5547329, 5710893] (unit: tons), while the predicted counterpart is [Low, R, Up] =[5736742, 6050701, 6252951] (unit: tons). An overall upward trend in freight volume for the next period could be observed. This suggests that when SVM is used in conjunction with FIG, it can effectively predict short-term freight volume trends.

### 3.3. Accuracy evaluation of the freight volume model

**Fig 8:**
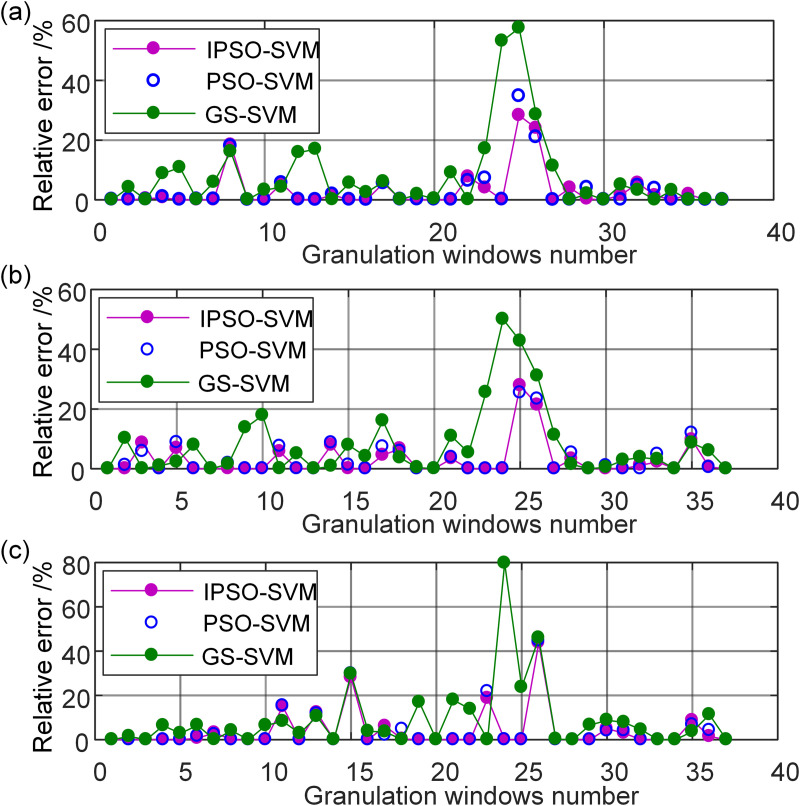
Prediction relative errors of granulated sets. (a) Low, (b) R, (c) Up.

To assess the accuracy of the IPSO-SVM algorithm in forecasting freight volume, the prediction relative errors were compared with those of grid search optimized SVM (GS-SVM) and PSO-SVM algorithms. Prediction relative errors of granulated sets are visually represented in Fig 8. The illustration clarified that the GS-SVM model exhibited the highest relative errors, succeeded by the PSO-SVM, whereas the IPSO-SVM demonstrated the lowest relative errors. Consequently, it could be inferred that, among the trio of models employed for freight volume trend prediction—GS-SVM, PSO-SVM, and IPSO-SVM—the IPSO-SVM model emerged as having superior predictive capability.‘

To thoroughly assess the predictive accuracy of each algorithm, the following metrics were employed: Mean Absolute Error (MAE), Root Mean Square Error (RMSE), Mean Absolute Percentage Error (MAPE), and Theil Inequality Coefficient (TIC). The definitions for these metrics are as follows:


$$\text{MAE}=\frac{1}{n}\sum_{i=1}^{n}\left|y_{i}-f(x_{i})\right|$$ (13)

$$\text{RMSE}=\sqrt{\frac{1}{n}\sum_{i=1}^{n}(y_{i}-f(x_{i}){)}^{2}}$$ (14)

$$\text{MAPE}=\frac{1}{n}\sum_{i=1}^{n}\left|\frac{y_{i}-f(x_{i})}{y_{i}}\right|\times 100\%$$ (15)

$$\text{TIC}=\frac{\sqrt{\sum_{i=1}^{n}(y_{i}-f(x_{i}){)}^{2}}}{\sqrt{\sum_{i=1}^{n}(y_{i}{)}^{2}}+\sqrt{\sum_{i=1}^{n}(f(x_{i}){)}^{2}}}$$ (16)

where *y*_*i*_ represents the actual value, while *f*(*x*_*i*_) denotes the predicted value, and *n* signifies the total number of sample data points analyzed.

Error comparison for SVM, PSO-SVM, and IPSO-SVM models are presented in Table 3.

**Table 3:** Errors Comparison Based on SVM, PSO-SVM, and IPSO-SVM Models.

Algorithms	Granulated sets	MAE	RMSE	MAPE (%)	TIC
GS-SVM	Low	153908	325785	8.86524	0.06970
	R	151053	267318	8.01183	0.05103
	Up	144162	275310	7.94269	0.04969
	Mean	149708	289471	8.27325	0.05681
[0pt] PSO-SVM	Low	88976	279614	4.15037	0.05945
	R	77321	182395	3.37242	0.03460
	Up	66587	169644	3.12983	0.03067
	Mean	77628	210551	3.55087	0.04157
[0pt] IPSO-SVM	Low	85039	274322	3.96114	0.05817
	R	66490	163713	2.98601	0.03104
	Up	66575	168592	3.01796	0.03026
	Mean	72701	202209	3.32170	0.03982

By evaluating the MAE metric, the IPSO-SVM model demonstrated a substantial improvement, with errors decreasing by 51.44% and 6.35% relative to the SVM and PSO-SVM algorithms, respectively. This trend was consistent across other error metrics: RMSE decreased by 30.15% and 3.96%, MAPE was reduced by 58.85% and 6.45%, and TIC dropped by 29.90% and 4.21%. These results collectively highlight the superior predictive performance of the IPSO-SVM model in forecasting railway freight volume time series, outperforming the GS-SVM and PSO-SVM counterparts.

In evaluating the prediction results of the prediction interval, this study adopted the following three key indicators:

(1) The maximum of absolute percentage error (${APE}_{\text{M}}$)

$${\text{APE}}_{\text{M}}=\max\left|\frac{f(x_{i})-y_{i}}{y_{i}}\right|,\;i=1,2,\ldots ,n$$ (17)

The smaller the value of ${APE}_{\text{M}}$, the less the deviation between the actual and predicted values, which signifies a more effective prediction outcome.

(2) Interval width (*W*): This metric was employed to avert the scenario where the prediction interval became unduly expansive due to an excessive focus on reliability, thereby impeding its ability to accurately depict uncertainty. A more compact prediction interval signifies a superior predictive outcome.

$$W=U(x_{i})-L(x_{i})$$ (18)

where *W* represents the interval width; *U*(*x*_*i*_) and *L*(*x*_*i*_) represent the upper and lower bounds of the prediction for the *i*-th sample, respectively.

(3) Relative Interval Width (*R*_*w*_): This metric quantifies the ratio of interval width to monthly average freight volume. It normalizes the absolute width *W* to the freight scale, enabling fair comparisons across periods and models. As freight volumes fluctuate, the same *W* implies different uncertainty; *R*_*w*_ thus assesses interval compactness for practical decision-making.

$$R_{w}=\frac{W}{V_{i}}\times 100\%$$ (19)

where *V*_*i*_ denotes the monthly average freight volume of the *i*-th sample period.

(4) Forecasting interval coverage proportion (FICP): This metric assesses the likelihood that the observed value resides within the forecasted intervals.

$$\text{FICP}=\frac{1}{n}\sum_{i=1}^{n}X_{i}\times 100\%$$ (20)

where *n* represents the number of predictions, if $X_{i}\in [L_{i},U_{i}]$, then *C*_*i*_=1; otherwise, *C*_*i*_=0. A higher value of FICP signifies that a greater proportion of actual values lie within the prediction interval, thereby indicating enhanced reliability and superior predictive performance of the interval forecast.

Table 4 presents the prediction intervals and result evaluations for freight volumes. Overall, the IPSO-SVM model exhibited superior performance in terms of the mean ${APE}_{\text{M}}$ for predicting the change interval of freight volume, with a value of only 5.03%, which is lower than the 5.14% and 9.49% achieved by the PSO-SVM and GS-SVM models, respectively. Additionally, the IPSO-SVM model achieved the smallest *R*_*w*_ of 8.53%, corresponding to the narrowest *W* of only 516,209 tons, compared to 8.82% for PSO-SVM and 22.01% for GS-SVM, while all three methods maintained a 100% coverage rate. These findings indicate that the IPSO-SVM freight volume prediction model achieves robust interval forecasting. It accurately captures both the change range and future trend of freight volumes.

**Table 4:** Prediction intervals and result evaluation for the freight volume.

Algorithms	Months	Actual values	Prediction Interval	${APE}_{𝐌}$ (%)	*W*	$R_{w}$ (%)	FICP (%)
GS-SVM	October	6165920	(4900745, 6058672, 6232929)	20.52%	1332184	22.01	100%
	November	6076187		2.50%			
	December	5911047		5.45%			
PSO-SVM	October	6165920	(5733075, 6053156, 6266738)	7.02%	533663	8.82	100%
	November	6076187		2.40%			
	December	5911047		6.01%			
IPSO-SVM	October	6165920	(5736742, 6050701, 6252951)	6.96%	516209	8.53	100%
	November	6076187		2.36%			
	December	5911047		5.78%			

## 4. Discussion

This study presents a novel FIG-IPSO-SVM framework for interval forecasting of railway freight volume. While the results quantitatively demonstrate its superiority over benchmark models, a deeper discussion is warranted to elucidate why this hybrid approach works, what broader implications it carries, and where its boundaries lie.

### 4.1. Interpretation of findings and hybrid model superiority

The superior performance of the FIG-IPSO-SVM model—evidenced by the lowest MAPE (3.32%) and the narrowest prediction interval width (516,209 tons)—can be attributed to the synergistic integration of its three components. First, the FIG preprocessing acts as a noise filter and trend extractor. By transforming raw noisy time-series into tri-granular representations (Low, R, Up), it effectively decouples the underlying trend from local volatility, providing a cleaner signal for subsequent model. Second, the IPSO algorithm addresses a critical weakness in SVM application—parameter sensitivity. The improved global search capability prevents the model from settling on suboptimal hyperparameters, which is a common pitfall for GS and standard PSO when dealing with complex, non-convex loss landscapes. This ensures a more thorough exploration of the hyperparameter space for SVM, leading to better generalization. Third, SVM was selected for its well-established strength in small-sample regression. Given only 114 monthly observations, SVM’s structural risk minimization principle provides strong theoretical guarantees against overfitting, which is often a challenge for data-intensive models. Together, these elements form a coherent pipeline that handles data uncertainty (FIG), optimizes model parameters (IPSO), and executes robust regression under sample constraints (SVM).

### 4.2. Methodological and practical implications

From a methodological perspective, this work validated the granulation-first paradigm for time-series forecasting under uncertainty. The key insight is that Fuzzy Information Granulation provides a structured representation framework that aligns naturally with the way human experts perceive trends and volatility. By design, the tri-granular structure (Low, R, Up) explicitly models the inherent uncertainty and range of variation within each temporal segment, shifting the analytical focus from precise but often unreliable point values to informative intervals. Consequently, this makes the model’s outputs inherently interpretable for decision-makers, bridging the gap between black-box predictions and operational logic.

Practically, the model shifts the focus from point prediction to interval management. For railway operators, knowing that future demand will fall within a interval of 516,209 tons with high confidence is more actionable than a single precise estimate that carries an unknown error. This corresponds to a relative width *R*_*w*_ of only 8.53%. Such precision directly supports robust optimization in resource scheduling, inventory buffering, and risk contract design, moving operations from reactive to proactive. The *R*_*w*_ metric directly quantifies the economic value of narrow prediction intervals—tighter intervals translate into lower buffer inventories, more precise capacity allocation, and higher decision-making efficiency.

Notably, the use of SVM is particularly justified in applications like railway freight forecasting, where data may be limited but the relationships are nonlinear and complex. The framework is inherently generalizable to other domains with similar characteristics—small samples, high noise, and nonlinear dynamics—such as energy demand forecasting, traffic flow prediction, or financial volatility modeling. The modular design also allows for the replacement of SVM with other regressors or the incorporation of additional exogenous variables in future extensions.

### 4.3. Limitations and boundary conditions

Despite its strengths, several limitations must be acknowledged. First, the model is trained and validated on data from a single freight center; its performance in regions with different economic structures, cargo mixes, or seasonal patterns remains to be tested, and cross-regional validation is needed to confirm broader applicability. Second, the current approach is univariate and does not explicitly incorporate external factors such as economic indicators, policy changes, or seasonal events, which could improve explanatory power. Third, the granulation scheme employs fixed parameters (e.g., a window size of 3). Although effective here, an adaptive or optimized granulation strategy might better capture varying temporal scales of volatility in different contexts.

Future research should therefore focus on:

(1) extending the framework to multivariate settings by including relevant exogenous variables.
(2) validating the model on multi-site and higher-frequency data to assess its robustness and scalability.
(3) exploring adaptive granulation strategies and alternative machine learning models (e.g., kernel-based methods or attention-based networks) for even greater predictive performance.

## 5. Conclusions

This study introduced a freight volume representation technique grounded in FIG and integrated it with the IPSO-SVM algorithm. A predictive model for freight volume trends was established utilizing the FIG-IPSO-SVM methodology, capable of forecasting both the intervals and trends of freight volume. Based on case simulations and analyses, two principal conclusions were drawn:

To address the inherent uncertainty, nonlinearity, and small-sample complexity of freight volume data, the proposed FIG-IPSO-SVM framework first employs FIG to granulate the time series into Low, R, and Up sets, reducing noise while preserving essential trend information. The IPSO-optimized SVM then performs granular-level regression, leveraging SVM’s kernel-based nonlinear modeling and strong generalization for small samples, with IPSO adaptively tuning parameters to enhance robustness. This integration synergistically tackles the core challenges, yielding highly accurate short-term freight volume predictions.

A comparative error analysis against the GS-SVM and PSO-SVM algorithms revealed that the IPSO-SVM-based freight volume model demonstrated superior performance, achieving minimal prediction errors across all granulated sets. Remarkably, it achieved the smallest mean ${APE}_{\text{M}}$for forecasting the subsequent period’s freight volume interval at 5.03%, coupled with the narrowest interval width (*W* = 516,209 tons) and the smallest relative width (*R*_*w*_=8.53%). These findings highlight the effectiveness and advantage of the FIG-IPSO-SVM freight volume trend model in accurately predicting future freight volume dynamics. This predictive approach offers significant potential for optimizing railway transport infrastructure and enhancing the strategic allocation of societal resources.

In summary, the FIG-IPSO-SVM framework offers a practical, uncertainty-aware tool for freight volume trend forecasting, with tangible implications for railway logistics planning and operational decision-making. Future work will focus on enhancing the model’s generality through the inclusion of external variables and validation across diverse operational scenarios.
